# Clinical and Treatment-Related Factors Associated With Recurrence After Definitive Radiotherapy for Early-Stage Glottic Squamous Cell Carcinoma: A Retrospective Cohort Study in Panama

**DOI:** 10.7759/cureus.111904

**Published:** 2026-07-01

**Authors:** Wilson Barrera, Gustavo A Chevasco-Champsaur, James M Quintero-Gill, Sophia Galbraith, Daniel Estribi, Francisco Palma-García, Rafael E Arauz, Gaspar Pérez Jiménez

**Affiliations:** 1 Radiation Oncology, Instituto Oncológico Nacional, Panama, PAN; 2 Department of Internal Medicine, Universidad de Panamá, Panama, PAN

**Keywords:** conformal, laryngeal neoplasms, laryngeal squamous cell carcinoma, larynx, progression-free survival, radiation dose hypofractionation, radiotherapy, recurrence

## Abstract

Background: Definitive radiotherapy is a standard larynx-preserving treatment for early-stage glottic squamous cell carcinoma. This study evaluated recurrence patterns and freedom from recurrence after definitive radiotherapy in Panama; explored baseline clinical, pathological, and treatment-related factors associated with recurrence; and analyzed initial post-treatment response as an early marker of subsequent recurrence risk.

Methods: We conducted a retrospective cohort study of adults diagnosed between 2013 and 2022 with T1-T2 N0 M0 glottic squamous cell carcinoma and treated with definitive radiotherapy at the National Oncology Institute of Panama. Time to recurrence, event-free survival, overall survival, and laryngeal preservation with oncologic control were evaluated. Freedom from recurrence was estimated using the Kaplan-Meier method, with deaths without prior recurrence censored. Survival outcomes were compared using log-rank tests and Cox proportional hazards models.

Results: Ninety-four patients were included: 79.8% had T1a, 13.8% had T1b, and 6.4% had T2 disease; 94.7% received three-dimensional conformal radiotherapy. An initial complete response was achieved in 86/93 patients (92.5%). With a median observed follow-up of 65.9 months (38.6-96.6), 17/94 patients (18.1%) developed recurrence: local recurrence occurred in 13.8% and regional recurrence in 4.3%, with no distant metastases. Freedom from recurrence at two and five years was 87.0% and 80.2%, respectively; five-year event-free survival was 70.4%, and five-year overall survival was 81.7%. Laryngeal preservation with oncologic control was achieved in 77/94 patients (81.9%). Partial response was associated with lower five-year freedom from recurrence compared with complete response (28.6% vs. 84.2%; p < 0.001). Five-year freedom from recurrence was 85.4% with hypofractionation and 78.5% with conventional fractionation (p = 0.483).

Conclusion: In this retrospective cohort with a median observed follow-up of 65.9 months, definitive radiotherapy achieved a high initial complete response rate, predominantly local recurrence patterns, and a five-year freedom from recurrence of 80.2%, with deaths without prior recurrence censored. Partial response identified a high-risk subgroup as an early post-treatment response marker. Hypofractionation showed a non-significant numerical trend toward higher five-year freedom from recurrence, but the study did not demonstrate superiority, equivalence, or non-inferiority between fractionation schedules.

## Introduction

Early-stage glottic squamous cell carcinoma has favorable oncologic outcomes when treated with curative intent, and treatment selection aims to combine tumor control, laryngeal preservation, and voice function. Definitive radiotherapy remains a standard organ-preserving approach for patients with T1-T2 N0 M0 disease, with high rates of local control and laryngeal preservation reported in historical and contemporary series [1,2]. Recurrence remains clinically relevant because it may require salvage treatment and compromise laryngeal preservation. Reported factors associated with radiotherapy failure include T stage, anterior commissure involvement, tumor extension, fraction size, total dose, hemoglobin level, smoking, and overall treatment time [3,4].

Radiotherapy fractionation for early glottic cancer has evolved from conventional schedules toward moderate hypofractionation or accelerated treatment. Prospective trials and meta-analyses suggest that shorter schedules may improve or maintain local control, probably through higher dose per fraction and reduced overall treatment time, although the magnitude of benefit may vary by stage, technique, and patient selection [5-8].

Published data from Central America on definitive radiotherapy outcomes for early-stage glottic cancer are limited. The primary objective of this study was to evaluate recurrence patterns and freedom from recurrence in patients diagnosed between 2013 and 2022 with T1-T2 N0 M0 glottic squamous cell carcinoma and treated with definitive radiotherapy at the Instituto Oncologico Nacional of Panama, using time to first documented recurrence as the endpoint and censoring deaths without prior recurrence. Secondary objectives were to explore baseline clinical, pathological, and treatment-related factors associated with recurrence, including T stage, anterior commissure involvement, hemoglobin level, smoking status, alcohol use, radiotherapy technique, and fractionation schedule. Initial post-treatment response was analyzed separately as an early response marker associated with the risk of subsequent recurrence. We hypothesized that adverse baseline anatomical factors, treatment-related variables, and incomplete initial response would be associated with lower freedom from recurrence; however, given the retrospective design and limited number of events, these analyses were considered exploratory.

## Materials and methods

Study design and population

**Figure 1:**
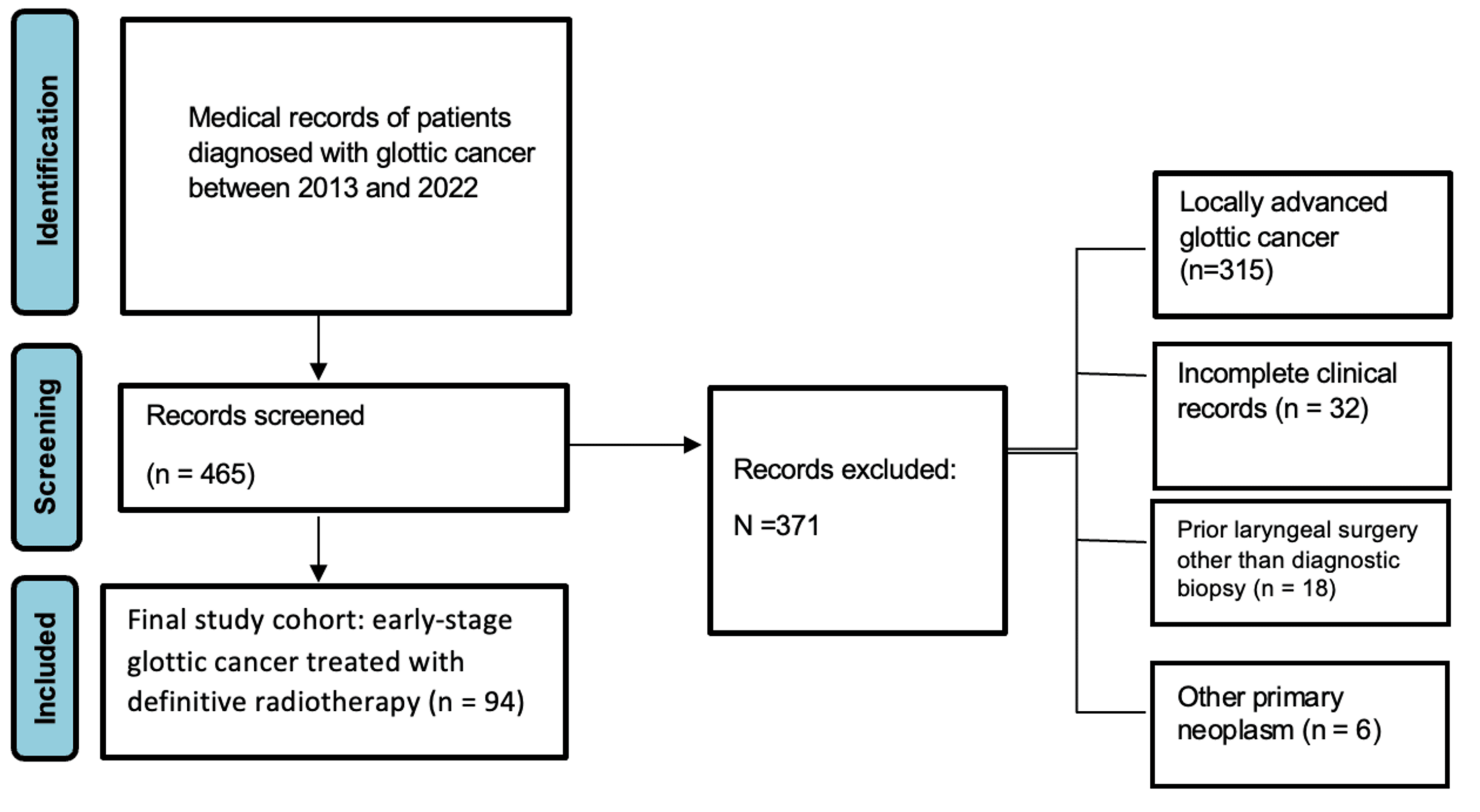
Patient selection flowchart A total of 465 medical records of patients diagnosed with glottic cancer between 2013 and 2022 were screened. Overall, 371 records were excluded: locally advanced glottic cancer (n = 315), incomplete clinical records (n = 32), prior laryngeal surgery before radiotherapy (n = 18), and other primary neoplasm (n = 6). The final study cohort included 94 patients with early-stage glottic cancer treated with definitive radiotherapy.

This retrospective cohort study included adults with biopsy-proven invasive T1-T2 N0 M0 glottic squamous cell carcinoma diagnosed between January 1, 2013, and December 31, 2022, and treated with definitive external-beam radiotherapy at the National Oncology Institute of Panama. Clinical staging was based on available clinical, endoscopic, and imaging records according to the AJCC 7th or 8th edition, depending on the treatment period. Radiotherapy initiation occurred between 2013 and 2023, as some patients diagnosed in late 2022 began definitive radiotherapy in 2023. Treatment-era analyses were therefore defined by the year of radiotherapy initiation. Patients were excluded if they received induction, concomitant, or adjuvant chemotherapy; had prior laryngeal surgery other than diagnostic biopsy; prior head and neck radiotherapy; no post-radiotherapy laryngeal assessment; follow-up <1 month; or insufficient data to evaluate recurrence (Figure 1).

Data collection and treatment variables

Data were retrospectively extracted from medical records, institutional registries, laryngoscopy reports, pathology reports, radiotherapy records, and treatment-planning systems. Extracted variables included demographic, clinical, pathological, treatment-related, recurrence, salvage, follow-up, and vital status data. For analytical clarity, variables were categorized as baseline/pre-treatment factors, treatment-related factors, and post-treatment response markers. Baseline and treatment-related factors were treated as exploratory variables potentially associated with recurrence, whereas initial response was analyzed as an early post-treatment marker rather than a baseline predictor.

Smoking status was classified as current, former, never, or passive exposure. Baseline hemoglobin was analyzed using the prespecified cutoff of ≤13 g/dL. Radiotherapy technique was classified as two-dimensional radiotherapy or three-dimensional conformal radiotherapy. Fractionation was classified as conventional when the dose per fraction was ≤2.0 Gy and hypofractionated when the dose per fraction was >2.0 Gy. BED10 was defined as the biologically effective dose using an α/β ratio of 10 Gy, and EQD2_10 as the equivalent dose in 2-Gy fractions using an α/β ratio of 10 Gy; both were calculated using the linear-quadratic model.

All patients were treated with definitive external-beam radiotherapy directed to the glottic larynx using institutional laryngeal field arrangements according to the treating radiation oncologist’s practice and the technology available during the treatment period. This study was not designed as a dosimetric analysis; therefore, target volume delineation, field borders, beam arrangement, organs-at-risk doses, dose-volume parameters, image-guidance procedures, and centralized plan quality review were not included as study variables. The treatment-related variables analyzed were those consistently available in the clinical and radiotherapy records: radiotherapy technique, total dose, number of fractions, dose per fraction, BED10, EQD2_10, and overall treatment time.

Outcomes

The primary outcome was recurrence after definitive radiotherapy, analyzed as both a binary outcome and a time-to-event endpoint. Recurrence was classified as local, regional, mixed locoregional, or distant. Recurrence ascertainment was based on the available clinical, endoscopic, imaging, pathology, and follow-up documentation. Histological confirmation was recorded when available. In cases without documented biopsy confirmation, recurrence was accepted when the treating team documented recurrent disease based on compatible endoscopic and/or imaging findings and subsequent management. The date of recurrence was defined as the earliest date on which recurrence was documented in the medical record.

Time to recurrence was defined as the time from radiotherapy initiation to the first documented recurrence. Patients without recurrence were censored at the date of last follow-up, and patients who died without prior recurrence were censored at the date of death. Freedom from recurrence was estimated using the Kaplan-Meier method. Because deaths without prior recurrence were censored, freedom from recurrence was interpreted as an estimate of documented recurrence among patients remaining under observation, rather than as a composite disease-control endpoint.

Event-free survival was defined as the time from radiotherapy initiation to recurrence or death from any cause, whichever occurred first. Overall survival was defined as the time from the initiation of radiotherapy to death from any cause. Locoregional control was defined as time to local, regional, or mixed locoregional recurrence. Laryngeal preservation with oncologic control was defined as absence of documented recurrence during follow-up. Post-treatment response and follow-up assessments were performed according to routine clinical practice and were not protocol-mandated. Initial response was classified as complete or partial according to the first documented post-radiotherapy clinical or endoscopic assessment. Follow-up information was extracted from oncology records, otolaryngology evaluations, endoscopy reports, imaging studies, pathology reports, and vital-status documentation when available.

Statistical analysis and ethics

Categorical variables were summarized as frequencies and percentages. Continuous variables were summarized as median, interquartile range, and range. Missing data were not imputed; analyses were performed using available data with variable-specific denominators. Freedom from recurrence, event-free survival, and overall survival were estimated using the Kaplan-Meier method and compared using the log-rank tests. Cox proportional hazards models were used to estimate hazard ratios and 95% confidence intervals. Because of the limited number of recurrence events, analyses were primarily univariable, and broad multivariable models were avoided to reduce overfitting. Clinical T stage was analyzed as T1a versus T1b/T2 because only six patients had T2 disease. A two-sided p <0.05 was considered statistically significant. Analyses were performed using IBM SPSS software (IBM Corp., Armonk, NY). The study was approved by the ethics committee under code EC-CBITPC-266. The database was anonymized and handled confidentially. The requirement for informed consent was waived because of the retrospective design.

## Results

A total of 465 medical records of patients diagnosed with glottic cancer between 2013 and 2022 were screened. Of these, 371 were excluded because of locally advanced glottic cancer (n = 315), incomplete clinical records (n = 32), prior laryngeal surgery before radiotherapy (n = 18), or other primary neoplasm (n = 6). The final cohort included 94 patients diagnosed between 2013 and 2022 with invasive early-stage T1-T2 N0 M0 glottic squamous cell carcinoma and treated with definitive radiotherapy (Figure 1).

Most patients were male (88.3%) and had T1a disease (79.8%). Anterior commissure involvement was documented in 28.1%. Baseline clinical and pathological characteristics are summarized in Table 1.

**Table 1:** Baseline clinical and pathological characteristics of the cohort Values are presented as n/N (%) unless otherwise indicated. Percentages were calculated using the number of evaluable patients for each variable. Hb, hemoglobin; IQR, interquartile range.

Variable	Value
Patients included	94 (100%)
Age at diagnosis	
Median (IQR), years	68 (61–76)
Range, years	37–92
Sex	
Male	83 (88.3%)
Female	11 (11.7%)
Clinical T stage	
T1a	75 (79.8%)
T1b	13 (13.8%)
T2	6 (6.4%)
Anterior commissure involvement	
Involved	25 (28.1%)
Not involved	64 (71.9%)
Alcohol use	
Documented alcohol use	64 (68.8%)
Smoking status	
Current smoker	5 (5.4%)
Former smoker	62 (66.7%)
Never smoker	20 (21.5%)
Passive exposure	6 (6.5%)
Baseline hemoglobin	
Available	91 (96.8%)
Hemoglobin, median (IQR), g/dL	14.4 (13.3–15.3)
Hb ≤13 g/dL	19 (20.9%)
Hb >13 g/dL	72 (79.1%)
Histological grade	
G1	34 (36.2%)
G2	50 (53.2%)
G3	3 (3.2%)

Three-dimensional conformal radiotherapy was used in 94.7% of cases. Conventional fractionation was delivered to 70 patients (74.5%) and hypofractionation to 24 patients (25.5%). Median BED10 was 84.0 Gy (81.6-84.0) with conventional fractionation and 80.52 Gy (80.52-80.52) with hypofractionation; median EQD2_10 was 70.0 Gy (68.0-70.0) and 67.1 Gy (67.1-67.1), respectively. Median treatment duration was 54 days (51-57) with conventional fractionation and 45.5 days (43.5-51.25) with hypofractionation. Treatment characteristics are presented in Table 2.

**Table 2:** Radiotherapy treatment characteristics by fractionation schedule BED10 (Biologically effective dose using an α/β ratio of 10 Gy), EQD2_10 (Equivalent dose in 2-Gy fractions using an α/β ratio of 10 Gy), IQR (interquartile range). Values are presented as n/N (%) or median (IQR) unless otherwise indicated. Conventional fractionation was defined as ≤2.0 Gy per fraction and hypofractionation as >2.0 Gy per fraction.

Variable	Conventional fractionation (n = 70)	Hypofractionation (n = 24)
Radiotherapy technique		
2D radiotherapy	5/70 (7.1%)	0/24 (0.0%)
3D conformal radiotherapy	65/70 (92.9%)	24/24 (100.0%)
Dose and fractionation		
Total dose, Gy, median (IQR)	70.0 (68.0–70.0)	66.0 (66.0–66.0)
Total dose, Gy, range	60.0–70.0	63.0–66.0
Number of fractions, median (IQR)	35 (34–35)	30 (30–30)
Number of fractions, range	30–38	28–30
Dose per fraction, Gy, median (IQR)	2.00 (2.00–2.00)	2.20 (2.20–2.20)
Dose per fraction, Gy, range	1.80–2.00	2.20–2.25
Biological dose parameters		
BED10, Gy, median (IQR)	84.0 (81.6–84.0)	80.5 (80.5–80.5)
BED10, Gy, range	72.0–84.0	77.2–80.5
EQD2_10, Gy, median (IQR)	70.0 (68.0–70.0)	67.1 (67.1–67.1)
EQD2_10, Gy, range	60.0–70.0	64.3–67.1
Treatment duration		
Overall treatment time, days, median (IQR)	54 (51–57)	45.5 (43.5–51.3)
Overall treatment time, days, range	36–85	38–73

Baseline characteristics according to fractionation schedule are presented in Table 3. No statistically significant differences were observed between conventional fractionation and hypofractionation groups for age, sex, clinical T stage, anterior commissure involvement, smoking status, alcohol use, baseline hemoglobin, histological grade, or radiotherapy technique. Treatment era showed a numerical imbalance, with hypofractionation more frequently used during 2013-2017 than 2018-2023, although this difference did not reach statistical significance.

**Table 3:** Baseline characteristics according to fractionation schedule Values are presented as n/N (%) unless otherwise indicated. Continuous variables are presented as median (IQR). Percentages were calculated using the number of evaluable patients for each variable. P-values are exploratory and were calculated using chi-square or Fisher exact tests for categorical variables and Mann–Whitney U tests for continuous variables. Conventional fractionation was defined as dose per fraction ≤2.0 Gy and hypofractionation as dose per fraction >2.0 Gy. Treatment era was defined according to the year of radiotherapy initiation; six patients diagnosed in 2022 initiated radiotherapy in 2023.

Variable		Conventional fractionation (n = 70)	Hypofractionation (n = 24)	p-value
Age at diagnosis, years, median (IQR)		68.5 (62.0–76.0)	66.5 (57.5–76.2)	0.477
Sex				0.141
Male		64/70 (91.4%)	19/24 (79.2%)	
Female		6/70 (8.6%)	5/24 (20.8%)	
Clinical T stage				0.903
T1a		56/70 (80.0%)	19/24 (79.2%)	
T1b		9/70 (12.9%)	4/24 (16.7%)	
T2		5/70 (7.1%)	1/24 (4.2%)	
Anterior commissure involvement				0.694
Involved		19/65 (29.2%)	6/24 (25.0%)	
Not involved		46/65 (70.8%)	18/24 (75.0%)	
Smoking status				0.487
Current smoker		4/70 (5.7%)	1/23 (4.3%)	
Former smoker		47/70 (67.1%)	15/23 (65.2%)	
Never smoker		16/70 (22.9%)	4/23 (17.4%)	
Passive exposure		3/70 (4.3%)	3/23 (13.0%)	
Alcohol use				0.804
Documented alcohol use		47/69 (68.1%)	17/24 (70.8%)	
No documented alcohol use		22/69 (31.9%)	7/24 (29.2%)	
Hemoglobin, g/dL, median (IQR)		14.4 (13.4–15.2)	14.6 (13.2–15.6)	0.496
Baseline hemoglobin category				0.995
Hb ≤13 g/dL		14/67 (20.9%)	5/24 (20.8%)	
Hb >13 g/dL		53/67 (79.1%)	19/24 (79.2%)	
Histological grade				0.382
G1		27/64 (42.2%)	7/23 (30.4%)	
G2		34/64 (53.1%)	16/23 (69.6%)	
G3		3/64 (4.7%)	0/23 (0.0%)	
Radiotherapy technique				0.324
2D radiotherapy		5/70 (7.1%)	0/24 (0.0%)	
3D conformal radiotherapy		65/70 (92.9%)	24/24 (100.0%)	
Treatment era				0.074
2013–2017		29/70 (41.4%)	15/24 (62.5%)	
2018–2023		41/70 (58.6%)	9/24 (37.5%)	

Initial response was evaluable in 93 patients; 86/93 (92.5%) achieved complete response, and 7/93 (7.5%) had partial response. During follow-up, 17/94 patients (18.1%) developed recurrence: 13 local recurrences (13.8%) and four regional recurrences (4.3%). No mixed locoregional recurrences or distant metastases were documented. Median time to recurrence among patients who developed recurrence was 15.0 months (10.3-30.9). Ten recurrent patients underwent total laryngectomy. Laryngeal preservation with oncologic control, defined as absence of documented recurrence, was 77/94 (81.9%). Initial response, recurrence pattern, salvage treatment, and global outcomes are shown in Table 4.

**Table 4:** Initial response, recurrence pattern, salvage treatment, and survival outcomes CR (Complete response), PR (Partial response), IQR (interquartile range), EFS (Event-free survival), RFS (Recurrence-free survival), OS (Overall survival). Values are presented as n/N (%) unless otherwise indicated. Event-free survival was defined as the time from radiotherapy initiation to recurrence or death from any cause. Recurrence-free survival was defined as the time from radiotherapy initiation to first recurrence; deaths without prior recurrence were censored. Laryngeal preservation with oncologic control was defined as the absence of documented recurrence during follow-up.

Variable	Value
Initial Response	
Evaluable initial response	93
CR	86 (92.5%)
PR	7 (7.5%)
Recurrence pattern	
Any recurrence	17 (18.1%)
Local recurrence	13 (13.8%)
Regional recurrence	4 (4.3%)
Mixed locoregional recurrence	0 (0%)
Distant metastasis	0 (0%)
Time to recurrence, median (IQR), months	15.0 (10.3-30.9)
Salvage treatment among recurrent patients	
Any salvage treatment	17 (100%)
Total laryngectomy	10 (58.8%)
Chemotherapy	2 (11.7%)
Palliative care	2 (11.7%)
Laryngeal preservation	
Total laryngectomy in the overall cohort	10 (10.6%)
Laryngeal preservation with oncologic control	77 (81.9%)
Follow-up and survival	
Observed follow-up, median (IQR), months	65.9 (38.6-96.6)
Deaths during follow-up	25 (26.6%)
Event-free survival	
5-years EFS	70.40%
Recurrence-free survival	
5-year RFS	80.20%
Overall survival	
5-year OS	81.70%

**Figure 2:**
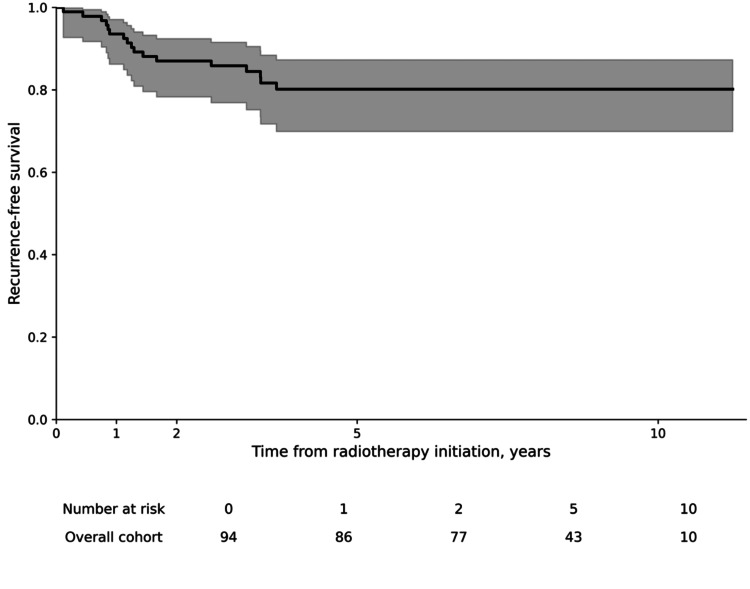
Recurrence-free survival after definitive radiotherapy Kaplan–Meier curve showing time from radiotherapy initiation to first documented recurrence, with deaths without prior recurrence censored. Freedom from recurrence was 87.0% at 2 years, 80.2% at 5 years, and 80.2% at 7 years.

Median observed follow-up was 65.9 months (38.6-96.6). Freedom from recurrence at 2, 5, and 7 years was 87.0%, 80.2%, and 80.2%, respectively, with deaths without prior recurrence censored (Figure 2). Five-year event-free survival was 70.4%, and 5-year overall survival was 81.7%. Partial response was associated with lower 5-year freedom from recurrence compared with complete response (28.6% vs. 84.2%; p < 0.001). Comparative time-to-event analyses are summarized in Table 5.

**Table 5:** Survival analyses according to clinical and treatment-related variables HR (hazard ratio), CI (confidence interval), PR (Partial response), CR (Complete response), EFS (Event-free survival), FFR (Freedom from recurrence), HF (Hypofractionation), CF (Conventional fractionation), Hb (hemoglobin).

Variable / comparison	Outcome	5-year estimate	HR	95% CI	p-value
Initial response					
PR vs CR	EFS	28.6% vs 74.6%	—	—	<0.001
PR vs CR	FFR	28.6% vs 84.2%	—	—	<0.001
Clinical T stage					
T1b/T2 vs T1a	FFR	70.7% vs 82.6%	—	—	0.335
Anterior commissure involvement					
Involved vs not involved	EFS	60.7% vs 73.6%	1.88	0.91–3.88	0.090
Involved vs not involved	FFR	71.3% vs 83.8%	—	—	0.375
Fractionation schedule					
HF vs CF	FFR	85.4% vs 78.5%	0.64	0.18–2.23	0.486
HF vs CF in T1a	FFR	89.2% vs 80.8%	—	—	0.552
Clinical exploratory variables					
Alcohol use vs no alcohol use	FFR	74.0% vs 96.3%	—	—	0.021
Current/former smoker vs non-smoker	FFR	72.7% vs 95.0%	—	—	0.049
Hb ≤13 g/dL vs >13 g/dL	FFR	82.8% vs 78.7%	—	—	0.665

Because only six patients had T2 disease, T stage was analyzed as T1a versus T1b/T2. Five-year freedom from recurrence was 82.6% for T1a and 70.7% for T1b/T2 (p = 0.335), with no statistically significant difference. Anterior commissure involvement showed a non-significant adverse signal for event-free survival from the start of radiotherapy: 60.7% versus 73.6% at five years in patients with and without involvement, respectively (p = 0.085). In univariable Cox analysis, anterior commissure involvement was associated with a non-significantly higher risk of event (HR, 1.88; 95% CI, 0.91-3.88; p = 0.090). Five-year freedom from recurrence was 71.3% versus 83.8%, respectively (p = 0.375). Five-year freedom from recurrence was 85.4% with hypofractionation and 78.5% with conventional fractionation (p = 0.483; HR, 0.64; 95% CI, 0.18-2.23; p = 0.486). In the T1a subgroup, 5-year freedom from recurrence was 89.2% with hypofractionation and 80.8% with conventional fractionation (p = 0.552).

In exploratory analyses, alcohol use was associated with lower five-year freedom from recurrence (74.0% vs. 96.3%; p = 0.021). Personal history of current or former smoking was also associated with lower freedom from recurrence compared with never smoking (72.7% vs. 95.0%; p = 0.049). Baseline hemoglobin ≤13 g/dL was not associated with significant differences in freedom from recurrence (82.8% vs. 78.7%; p = 0.665).

## Discussion

Definitive radiotherapy is an established organ-preserving treatment for early-stage glottic squamous cell carcinoma. In this retrospective cohort of 94 patients with T1-T2 N0 M0 disease diagnosed between 2013 and 2022 and treated at a national cancer center in Panama, initial complete response was frequent, recurrences were predominantly local, and no distant metastases were documented. Five-year freedom from recurrence, estimated with deaths without prior recurrence censored, was 80.2%, five-year event-free survival was 70.4%, and five-year overall survival was 81.7%. These findings support the effectiveness of definitive radiotherapy in routine clinical practice while also identifying clinically relevant areas of uncertainty related to response assessment, recurrence risk, treatment selection, and competing mortality.

Our outcomes are broadly consistent with international data. In the Dutch national cohort by Linden et al., five-year overall survival, relative survival, and recurrence-free survival were 75%, 88%, and 86%, respectively, and 6% required laryngectomy [2]. Classic radiotherapy series reported five-year local control ranging from approximately 85-94% for T1 and 70-80% for T2 disease [4,9-10]. In our cohort, crude local and locoregional control were 86.2% and 81.9%, respectively, which fall within these reported ranges. However, crude control rates, freedom from recurrence with death censored, and recurrence-free survival definitions are not directly interchangeable across studies.

The lack of a statistically significant association between the grouped T stage and freedom from recurrence should be interpreted with caution. Most patients had T1a disease, and only six had T2 tumors. Prior studies have shown inferior local control for T2 disease, especially in the presence of impaired vocal cord mobility, subglottic extension, or greater tumor burden [4,9,11]. Therefore, our results do not refute the prognostic value of T stage; rather, they reflect limited power to evaluate stage-related outcomes and the absence of detailed anatomic variables.

Initial response after radiotherapy was a clinically relevant marker of subsequent recurrence risk. Patients with partial response had markedly lower five-year freedom from recurrence than those with complete response, 28.6% versus 84.2%. Because response was assessed after treatment, it should not be interpreted as a baseline prognostic factor or as a pretreatment predictor. Instead, it represents an early post-treatment response marker. Although the partial-response subgroup was small, the magnitude of difference supports close endoscopic surveillance, early reassessment, and a low threshold for biopsy when persistent disease is suspected.

Anterior commissure involvement showed an adverse but non-significant signal. In event-free survival analysis, anterior commissure involvement was associated with a non-significantly higher risk of an event in a univariable Cox analysis. Although this association did not reach statistical significance, the confidence interval was wide and compatible with a clinically meaningful increase in event risk. This is consistent with reports identifying anterior commissure involvement as a potential risk factor for radiotherapy failure [10,12]. However, the evidence remains heterogeneous, with some institutional series failing to show a significant association [3,9,11,13]. Differences in anatomic definition, endoscopic assessment, tumor burden, field design, margins, and dosimetric coverage may contribute to this variability. International contouring guidelines emphasize adequate coverage of the anterior commissure and, when appropriate, the anterior contralateral vocal cord [14]. Our study did not include centralized plan review, detailed dosimetric assessment, or failure mapping; therefore, technical contributors to recurrence, including anterior commissure target coverage or marginal failure, could not be evaluated.

Five-year freedom from recurrence was numerically higher with hypofractionation than with conventional fractionation, both overall and in the T1a subgroup, although differences were not statistically significant. These findings are directionally consistent with prospective and pooled evidence. Yamazaki et al. showed improved five-year local control with 2.25 Gy per fraction versus 2.0 Gy per fraction, 92% versus 77% [5]. KROG-0201 showed a numerical advantage for hypofractionation, particularly in T1a tumors [6]. JCOG0701A3 reported comparable global outcomes between accelerated and standard fractionation, with lower cumulative local progression in the accelerated arm [7]. Benson et al. found improved local control with moderate hypofractionation, without overall survival benefit [8].

However, the present study was not designed or powered to establish superiority, equivalence, or non-inferiority between fractionation schedules. Treatment allocation was not randomized. Because fractionation schedule was selected according to clinical practice rather than random assignment, differences in treatment era, physician preference, institutional practice, patient selection, and logistical factors may have influenced which patients received hypofractionation. Baseline characteristics according to fractionation schedule are provided in Table 3. Although no statistically significant baseline differences were observed, treatment era showed a numerical imbalance, and residual treatment-selection bias cannot be excluded. Therefore, the hypofractionation findings should be interpreted as observational and exploratory. The findings suggest no obvious adverse signal in this cohort, but these do not prove that hypofractionation is equivalent or superior to conventional fractionation.

The radiobiological rationale for hypofractionation is based on higher dose per fraction and shorter overall treatment time. In squamous tumors, treatment prolongation may reduce biological effect through accelerated repopulation, and time-corrected BED models have been proposed to account for this loss [5,15]. In our cohort, BED10 was similar between patients with and without recurrence, and no clear association between additional treatment prolongation and locoregional failure was demonstrated. Given the low number of events, predominance of T1a disease, and few T2 tumors, this negative finding should not be interpreted as evidence that treatment time is irrelevant.

Freedom from recurrence should also be interpreted in light of the censoring strategy. Deaths without prior recurrence were censored in the freedom-from-recurrence analysis; therefore, this endpoint estimates documented recurrence occurrence among patients remaining under observation and should not be interpreted as a composite disease-control endpoint. Event-free survival was reported separately to account for death as an event. We did not perform competing-risk cumulative incidence analyses; therefore, freedom from recurrence may overestimate disease control compared with endpoints that treat death as a competing event or as part of a composite endpoint, particularly in an older cohort with non-negligible all-cause mortality.

Laryngeal preservation should be interpreted conservatively. Although total laryngectomy was documented in 10.6% of the overall cohort, the absence of laryngectomy does not necessarily indicate successful functional laryngeal preservation, because some recurrent patients were not surgically salvaged or had no salvage information available. We therefore reported laryngeal preservation with oncologic control, defined as absence of documented recurrence, achieved in 77/94 patients (81.9%). This is an anatomic-oncologic endpoint, not a functional one; contemporary assessment should also include toxicity, voice quality, swallowing function, and patient-reported outcomes [16].

Exploratory clinical variables should also be interpreted with caution. Alcohol use and personal smoking history were associated with lower freedom from recurrence; however, these associations were not adjusted in a robust multivariable model, alcohol exposure was not quantified, and smoking data lacked pack-years and longitudinal cessation status. Therefore, these findings should be considered hypothesis-generating rather than causal or independently prognostic. Baseline hemoglobin ≤13 g/dL was not associated with worse freedom from recurrence, although the limited number of events restricts inference.

The main limitations are the retrospective, single-center design, limited sample size 17 recurrences, predominance of T1a disease, and few T2 tumors. These factors increase the risk of selection bias, residual confounding, limited statistical power, and false-negative findings in subgroup analyses. Comparisons between conventional fractionation and hypofractionation were observational and may be affected by treatment-selection bias, including differences in treatment era, physician preference, institutional practice, patient selection, and logistical factors. Several variables depended on clinical documentation; recurrence confirmation was based on available pathology, endoscopic, imaging, and clinical records, and response assessment and follow-up were not protocol-standardized. Because this was not a dosimetric study, detailed target volume delineation, field design, dose-volume parameters, image guidance, and centralized plan quality review were not available for uniform analysis. Therefore, technical contributors to recurrence, including target coverage of the anterior commissure or marginal failures, could not be assessed. Smoking and alcohol exposure were not quantified, and toxicity, voice quality, swallowing function, and patient-reported outcomes were not systematically evaluated.

Despite these limitations, the study provides real-world data from a region with limited published evidence on definitive radiotherapy outcomes for early-stage glottic cancer. Strengths include a homogeneous glottic T1-T2 N0 M0 cohort, long observed follow-up, complete treatment dates for time-to-event analyses, and evaluation of clinically relevant outcomes including recurrence pattern, salvage treatment, freedom from recurrence, event-free survival, overall survival, and laryngeal preservation with oncologic control.

## Conclusions

In conclusion, definitive radiotherapy provided effective disease control in this retrospective cohort, with five-year freedom from recurrence of 80.2% and laryngeal preservation with oncologic control in 81.9% of patients. Recurrences were predominantly local. Partial response was associated with a substantially higher risk of recurrence and should be interpreted as an early post-treatment response marker rather than as a baseline prognostic factor. Hypofractionation showed a non-significant numerical trend toward higher five-year freedom from recurrence, but these data do not establish superiority, equivalence, or non-inferiority. Given the observational design, the limited number of recurrences, the small subgroups, and the absence of a competing-risk cumulative incidence analysis, all subgroup findings should be interpreted as exploratory.
